# Multiple Risk Assessment of Heavy Metals in Surface Water and Sediment in Taihu Lake, China

**DOI:** 10.3390/ijerph192013120

**Published:** 2022-10-12

**Authors:** Jiwei Yang, Fuhong Sun, Hailei Su, Yanru Tao, Hong Chang

**Affiliations:** 1 State Key Laboratory of Environmental Criteria and Risk Assessment, Chinese Research Academy of Environmental Sciences, Beijing 100012, China; 2 College of Environmental Sciences & Engineering, Beijing Forestry University, Beijing 100083, China

**Keywords:** heavy metals, ecological risk, health risk, surface sediments

## Abstract

Taihu Lake is the third-largest freshwater lake in eastern China. The contamination of heavy metals (HMs) in Taihu Lake resulting from rapid economic development and population growth has raised significant concerns in recent years. In this study, the contents and spatial distributions of eight typical HMs (Hg, Cr(VI), As, Cd, Cu, Ni, Pb, and Zn) in the fresh surface water and sediments from Taihu Lake were investigated. The potential ecological and health risks posed by HMs were evaluated using multiple assessment methods. Risk quotients were used to assess the ecological risks of HMs, and chronic risk quotients of Cu, Ni, and Pb (>1.0) were found in the surface water of Taihu Lake. According to the geo-accumulation index (I*_geo_*) and pollution load index (PLI) values, the lake sediments exhibited moderate risks of Cd and Hg. In general, the sediments were moderately contaminated by HMs based on the average risk index (RI < 300). Spatially, a high ecological risk posed by the HMs existed in the sediments of northern Taihu Lake (RI > 300), while the sediments in the southwestern and eastern regions had moderate risk levels. The non-carcinogenic risk levels of Hg, Cd, Cu, and Zn were acceptable based on the exposure characteristics of residents living around Taihu Lake. The carcinogenic risk levels of Cr(VI), As, Pb, and Ni through drinking water were acceptable. However, the ingestion of Cr(VI), As, and Ni through drinking water and fish consumption may pose certain health risks. Therefore, the levels of toxic metals, in particular, Cr(VI), As, and Ni, in edible organisms should be monitored periodically and controlled to alleviate the potential carcinogenic risks through food ingestion. Our work provides valuable information concerning the ecological risk distribution of HMs in Taihu Lake, which is essential for protecting the safety of aquatic organisms and human health and minimizing HM pollution in the lake.

## 1. Introduction

Heavy metal (HM) contamination in the water and sediments of rivers and lakes has attracted worldwide attention. Compounds such as Mercury (Hg), Arsenic (As), Cadmium (Cd), Chromium (Cr), and Copper (Cu) are examples of contaminating HMs [1,2]. Industrial wastewater discharge, household and public sewage, the mining industry, waste incineration, and aquaculture all result in the output of HMs into the environment. Such toxic metals have been detected in various environmental media, including water, sediments, and aquatic organisms [3,4]. The contents of typical metals in the surface sediments of the Yalu River Estuary and its adjacent waters were measured as Cr 19.9–90.9 mg∙kg^−1^, Ni 8.39–39.7 mg∙kg^−1^, Cu 2.9–193.2 mg∙kg^−1^, Zn 48.7–183.2 mg∙kg^−1^, Cd 0.1–0.5 mg∙kg^−1^, Pb 19.3–48.7 mg∙kg^−1^, As 2.3–15.9 mg∙kg^−1^, and Hg 15.2–302.8 mg∙kg^−1^ [5]. Sediment is an important reservoir of metals in aqueous environments. HMs adhered to sediment not only affect the safety of aquatic organisms and human health but also can be re-released under certain external conditions to become a secondary source of pollution. Therefore, more attention should be paid to the concentrations of metals in sediments and the risks that this poses [6].

HMs are persistent, highly toxic, and bioaccumulative. They are generally incorporated, precipitated, and enriched in sediments and some aquatic organisms [7]. Exposure can cause damage to aquatic organisms and the human body [8]. For example, As is closely related to the occurrence and symptoms of cardiovascular disease, hypertension, liver disease, and tumors [9]. HM pollution can also cause various histopathological changes in fish. It has been reported that the species mean acute toxicity value (SMAV) of Cu for *Crucian carp* was 208.0 μg∙L^−1^, and the chronic SMAV was 70.0 μg∙L^−1^ [10]. China and the United States Environmental Protection Agency (USEPA) both list Hg, As, Cd, Cr, Cu, Zn, and Pb as priority pollutants.

As the third-largest freshwater lake in China, Taihu Lake has become a diverse ecoregion with numerous industrial, agricultural, and aquacultural activities. The Taihu Lake basin is one of the most developed areas in China, with a population of 67.55 million. It is also an important drinking water source for native residents. Therefore, it is vital to protect the safety of surface water from pollution, which can influence the health of aquatic ecosystems and humans. The HM pollution and severe eutrophication in Taihu Lake have raised increasing concern from scientists and the local government [11,12]. Previous studies have focused on the exposure characteristics and source identification of HM in surface water and sediments of Taihu Lake [11,13]. Our previous investigation indicated that the number of fish, zooplankton, macrophyte, and benthic invertebrate species in Taihu Lake has declined significantly since 1980 [14]. Water pollution, caused by a variety of chemicals generated during anthropogenic activities, is a major threat to aquatic life. Risk assessments are typically used to assess the potential harm of specific pollutants to the ecosystem and human health at concentrations detected in the environment; such assessments play an important role in regulatory decisions. Therefore, it is necessary to investigate the potential ecological and human health risks of HMs in different environmental media to guide the prevention and control of HMs pollution in Taihu Lake. HMs are often introduced into the environment as mixtures. However, the information on the ecological and health risk of combined metal pollution in Taihu Lake is still limited. Additionally, previous studies concentrated on the northern and eastern parts of Taihu Lake, such as Meiliang Bay, Gongshan Bay, Zhushan Bay, East Taihu Bay, and so on. The overall assessment risk of HMs in surface water and sediment of the whole of Taihu Lake is more informative for policymakers to establish pollution control measures. 

The main objective of this study was to investigate the distributions of eight typical HMs (Hg, Cr(VI), As, Cd, Cu, Ni, Pb, and Zn) in the surface water and sediments of the whole of Taihu Lake, China, and assess the potential ecological and human health risks of HMs via multiple analytical and assessment approaches. Risk quotients (RQs) were used to assess the ecological risk of HMs in surface water. The geo-accumulation index (I*_geo_*), the pollution load index (PLI), and the potential ecological risk index (RI) were used to assess the degree of contamination and ecological risks of the HMs in sediments of Taihu Lake. Furthermore, the potential health risks associated with drinking water and fish consumption were examined according to the US EPA methods. The in-depth results regarding risk assessments of HMs in the surface water and sediments can be used to improve the environmental management of HMs in Taihu Lake.

## 2. Materials and Methods

### 2.1. Study Area and Sample Collection

This study investigated the concentrations of eight HMs in surface waters and sediments in Taihu Lake. The lake is located in the lower reaches of the Yangtze River Basin. During the Mesozoic, large-scale granite invaded the basin of Taihu Lake, and the famous Suzhou granite formed. The elevation of the southwestern mountain is higher than the northeast region. The contents of organic matter and carbonate are relatively low in the sediment of Taihu Lake, which has fewer effects on the contents of metal [6]. Taihu Lake is the third largest freshwater lake in China, with a surface area of 2400 square kilometers and a drainage area of 36,895 square kilometers. Taihu Lake is a typical shallow lake with a maximum depth of less than 3.0 m and an average depth of 1.9 m. Taihu Lake has abundant rainfall, with an average annual precipitation of 1100 mL. It belongs to the subtropical monsoon climate zone. The Taihu Lake basin is one of the most developed industrial and agricultural areas in China. It is also an important water source, aquaculture area, and aquatic habitat. Along with the rapid economic development and intensive use of water resources, the water quality of Taihu Lake has deteriorated and suffered from eutrophication and cyanobacterial blooms. 

**Figure 1:**
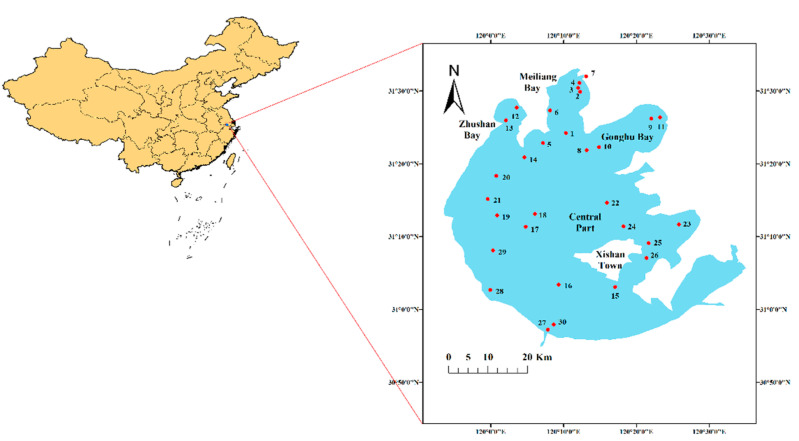
Map of sampling sites in Taihu Lake.

A total of 30 fresh surface water and sediment samples were collected from Taihu Lake in October 2019. The sampling sites are shown in Figure 1. At each sampling location, five independent water/sediment samples were collected and pooled to obtain one composite water/sediment sample. The sediment samples (upper 20–30 cm) were collected using a stainless grab sampler. The water samples were acidified and extracted immediately, and the sediment samples were stored at −20 °C until further analysis. The sediment was air-dried and then ground through a 0.15 mm sieve to remove the visible plant debris and stones and to homogenize the sample. The preparation process avoided contamination and the loss of material.

### 2.2. Analyses of HM Concentrations in the Surface Water and Sediments

The pretreatment of the water samples and the quantification of HMs were carried out according to the standardized methods from the Ministry of Ecology and Environment (MEE) of China with minor modifications [15]. The water samples for the detection of HMs were acidified with HNO_3_ to a pH < 2 and then stored at 4 °C. The acidified samples were placed in a cool container and immediately delivered to the laboratory. Concentrations of Cd, Cu, Pb, Zn, and Ni in the surface water of Taihu Lake were measured by inductively coupled plasma–mass spectrometry (ICP-MS, Elan 6000, Perkin Elmer, Waltham, MA, USA). As the concentrations in the water samples were analyzed by hydride generation atomic fluorescence spectrometry (HG-AFS) using a Millennium Excalibur system (PSA 10.055. P S Analytical Ltd., Orpington, Kent, UK). Hg concentrations in water samples were detected by vapor generation–atomic fluorescence spectrometry using a Millennium Merlin system (PSA 10.025. P S Analytical Ltd., Orpington, Kent, UK). The Cr(VI) concentrations in the water samples were determined by flow injection analysis (FIA) and the diphenyl carbazide spectrometric method [16].

In the laboratory, the pretreatment of the sediment samples for Cd, Cu, Pb, Zn, Ni, Hg, and As was carried out according to the standardized methods from the MEE of China with minor modifications [17]. Briefly, 6 mL of a 3:1 mixture of concentrated HCl and HNO_3_ was added to 0.1 g of each sample, followed by digestion in a microwave sample preparation system (Mini WAVE, SCP Science, Quebec, QC, Canada) for 1 h. The concentrations of Cd, Cu, Pb, Zn, and Ni in the digested solutions were analyzed using ICP-MS, and the contents of Hg and As were detected using atomic fluorescence spectrometry (AFS). Cr(VI) concentrations in sediment samples were determined by flame atomic absorption spectrometry after alkaline digestion [18]. The samples were analyzed within a week. All of the chemical reagents used were of super-pure grade. The experimental glassware was precleaned by soaking in 15% HNO_3_ (*w*/*w*) for at least 24 h, followed by soaking and rinsing with ultrapure water prior to use.

Quality control for the sediment samples was achieved by the use of certified reference materials (GBW07312) produced by the Institute of Geophysical and Geochemical Exploration, Chinese Academy of Geological Sciences. Analytical reagent blanks were prepared with each batch of digested samples and then analyzed in the same way for background correction. Each sample was measured in triplicate to assess the accuracy and precision. The analytical results for the reference materials were within 10% variability, and the relative standard deviations (RSDs) for the triplicate samples were less than 10%. The detection limits were 0.01 mg∙kg^−1^ for Cd, 0.05 mg∙kg^−1^ for Cu, 0.1 mg∙kg^−1^ for Pb, 0.2 mg∙kg^−1^ for Zn, 0.4 mg∙kg^−1^ for Ni, 0.001 mg∙kg^−1^ for Hg and As, and 0.25 mg∙kg^−1^ for Cr(VI). The recovery rates of Cd, Cr(VI), Cu, Pb, Zn, Ni, Hg and As were 96.8–105.3%, 91.1–105.2%, 94.7–106.8%, 95.1–106.3%, 93.2–105.4%, 94.6–107.6%, 97.5–103.1%, and 97.5–103.8%, respectively.

### 2.3. Ecological Risk Assessment of HMs in Surface Water

The ecological risks of HMs in the fresh surface water were assessed by the method of risk quotients (RQs). The RQs were calculated as the ratio of the measured concentrations of individual metals to the aquatic life criteria [19]. The acute and chronic aquatic life criteria of HMs for Chinese native freshwater organisms were retrieved from peer-reviewed literature and official reports. The acute and chronic RQs were calculated in this study. The RQs were categorized as follows: (1) RQ ≥ 1.00, high ecological risk; (2) 0.10 ≤ RQ < 1.00, medium ecological risk; and (3) RQ < 0.1, low ecological risk.

### 2.4. Ecological Risk Assessment of HMs in Sediments

#### 2.4.1. Geo-Accumulation Index (I*_geo_*) Method

The I*_geo_* method proposed by Müller is commonly used to assess the degree of contamination by HMs in sediments [20]. The index quantitatively expresses the degree of pollution by individual metals in particular locations as the ratio of the measured concentration to the geochemical background concentration. The I*_geo_* values were calculated using Equation (1):

(1) $$I_{geo}=\log_{2}(\frac{C_{n}}{1.5\times B_{n}})$$

where C*_n_* is the measured concentration of an individual metal in a sediment sample (mg∙kg^−1^); 1.5 is a correction coefficient considering that the diagenesis might cause background value fluctuation, and B*_n_* is the background value of the individual metal. In this study, the values of B*_n_* for the eight metals were adopted according to the previous study [21]. The I*_geo_* values were categorized as follows: (1) I*_geo_* < 0, pollution level 0, indicating no pollution; (2) 0 ≤ I*_geo_* < 1, pollution level 1, indicating no to moderate pollution; (3) 1 ≤ I*_geo_* < 2, pollution level 2, indicating moderate pollution; (4) 2 ≤ I*_geo_* < 3, pollution level 3, indicating moderate to heavy pollution; (5) 3 ≤ I*_geo_* < 4, pollution level 4, indicating heavy pollution; (6) 4 ≤ I*_geo_* < 5, pollution level 5, indicating heavy to extremely heavy pollution; and (7) I*_geo_* ≥ 5, pollution level 6, indicating extremely heavy pollution [22].

#### 2.4.2. Pollution Load Index (PLI) Method

The PLI method is commonly used for evaluating the combined effects of the contaminants [23,24], and this index was used to measure the eight HMs in the present work. The smaller the index value, the lower the pollution degree.

(2) $$\mathrm{PI}=\frac{C_{n}}{B_{n}}$$

(3) $$\mathrm{PLI}=\sqrt[{8}]{{\mathrm{PI}}_{Hg}\times {\mathrm{PI}}_{Cr(\mathrm{VI})}\times {\mathrm{PI}}_{As}\times {\mathrm{PI}}_{Cd}\times {\mathrm{PI}}_{Cu}\times {\mathrm{PI}}_{Ni}\times {\mathrm{PI}}_{Pb}\times {\mathrm{PI}}_{Zn}}$$

Here, C*_n_* is the measured concentration of an individual metal in a sediment sample (mg∙kg^−1^); B*_n_* is the background value of the individual metal; PI is the pollution load index of an individual metal; and PLI is the pollution load index of eight HMs at a given sample site. The PI and PLI are divided into four categories, as shown in Table 1.

#### 2.4.3. Potential Ecological Risk Index (RI) Method

The RI method was also applied to calculate the potential ecological risk of a single metal and the sum of the eight HMs in the sediments of Taihu lake [25].

(4) $${\mathrm{EI}=T}_{i}\times {\mathrm{PI}}_{i}$$

(5) $$\mathrm{RI}=\sum_{i=1}^{n}\mathrm{EI}$$

Here, EI is the potential risk of an individual metal; T*_i_* is the toxicity effect coefficient with values of Hg = 40, Cr(VI) = 2, As = 10, Cd = 30, Cu = 5, Ni = 5, Pb = 5, and Zn = 1; RI is the ecological risk of eight HMs in sediments. The categories of the EI and RI values are shown in Table 2.

### 2.5. Human Health Risk Assessment for Metal in Surface Water

#### 2.5.1. Non-Carcinogenic Health Risk from Drinking Water and Fish Consumption

Drinking water and fish consumption are the most important exposure routes for metal ingestion from surface water for residents living near Taihu Lake. The non-carcinogenic health risks for a single metal (Hg, Cd, Cu, and Zn) associated with drinking water or fish consumption were assessed using hazard quotients (HQs) in the present study. The non-carcinogenic health risk is defined as the ratio of the lifetime average daily dose (ADD) to the reference dose (RfD) [26]. An HQ of greater than 1.0 indicates possible harm to human health.

(6) $$\mathrm{HQ}\;\mathrm{from}\;a\;\mathrm{single}\;\mathrm{exposure}\;\mathrm{routine}=\frac{\mathrm{ADD}}{\mathrm{RfD}}$$

(7) $${\mathrm{THQ}=\mathrm{HQ}}_{W}+{\mathrm{HQ}}_{F}\;$$

where ADD is the average daily dose from a single route (mg∙kg^−1^∙d^−1^); RfD is the reference dose of a single metal (mg∙kg^−1^∙d^−1^); HQ*_W_* is the hazard quotients from drinking water; HQ*_F_* is the hazard quotients from fish consumption; THQ is the total hazard quotient for an individual metal resulting from drinking water and fish consumption. 

In this study, the total non-carcinogenic THQ (TTHQ) was calculated as the sum of the individual metal THQ values according to the method of the U.S. EPA [26,27]:


TTHQ = HQ*_Hg_* + HQ*_Cd_* + HQ*_Cu_* + HQ*_Zn_* (8)

The lifetime average daily exposure dose (ADD) of HMs through the consumption of drinking water or fish consumption was calculated according to the methods of the U.S. EPA [25,26].

(9) $${\mathrm{ADD}}_{W}=\frac{C_{W}\times \mathrm{DI}\times \mathrm{EF}\times \mathrm{ED}}{\mathrm{BW}\times \mathrm{AT}}$$

(10) $${\;\mathrm{ADD}}_{F}=\frac{C_{F}\times \mathrm{FI}\times \mathrm{EF}\times \mathrm{ED}}{\mathrm{BW}\times \mathrm{AT}}\;$$

(11) $$C_{F}=\mathrm{BCF}\times C_{W}$$

where ADD*_W_* is the average daily exposure dose from drinking water (mg∙kg^−1^∙d^−1^); ADD*_F_* is the average daily exposure dose from fish consumption (mg∙kg^−1^∙d^−1^); C*_W_* is a single metal concentration in the water (mg∙L^−1^); DI is the drinking water intake (mL∙day^−1^); EF is the exposure frequency (365 days∙year^−1^); ED is exposure duration (70 years); BW is the body weight for Chinese residents (kg); AT is the averaging exposure time for non-carcinogens (365 days∙year^−1^ × number of exposure years); FI is the fish consumption of native residents (g∙day^−1^); C*_F_* is the concentration of a single metal in fish (mg∙L^−1^); BCF is the bioconcentration factor (L∙kg^−1^). The exposure parameters, including DI, BW, and FI, for the residents living around Taihu Lake, were adopted. A higher ADD value indicates that the health risks posed by HMs are higher.

#### 2.5.2. Carcinogenic Health Risk from Drinking Water and Fish Consumption

The carcinogenic risk is defined as the incremental probability that an individual will suffer cancer due to exposure to certain contaminants during one’s whole lifetime. The carcinogenic health risks ® for single metals (Cr(VI), As, Ni, and Pb) associated with drinking water or fish consumption were calculated as follows [26,27,28].

(12) R from a single exposure route=SF × ADD

(13) $${\mathrm{TR}=R}_{W}+R_{F}\;$$

where SF is the oral slope factor, ([mg∙kg^−1^∙day^−1^]^−1^); R*_W_* is the carcinogenic health risks from drinking water; R*_F_* is the carcinogenic health risks from fish consumption; TR is the total carcinogenic risk for an individual metal. A cancer risk value R between 10^−6^ and 10^−4^ means an acceptable risk level. Otherwise, there could be a carcinogenic risk to human health.

The total carcinogenic risk (TR) was calculated as the sum of individual metal R values according to the method of the U.S. EPA [27]:


TR = R*_Cr(VI)_* + R*_As_* + R*_Pb_* + R*_Ni_* (14)

### 2.6. Data Analyses

The descriptive statistics (including the mean, median, standard deviation, and coefficient of variation (CV)) were calculated to describe the HM concentrations in the sediments and water using Origin 10.5 software. The spatial distributions of the HM concentrations and the values of I*_geo_* and RI were visualized with ArcGIS 10.3 software.

## 3. Results and Discussion

### 3.1. Concentrations and Distribution of HMs in the Fresh Surface Water

**Figure 2:**
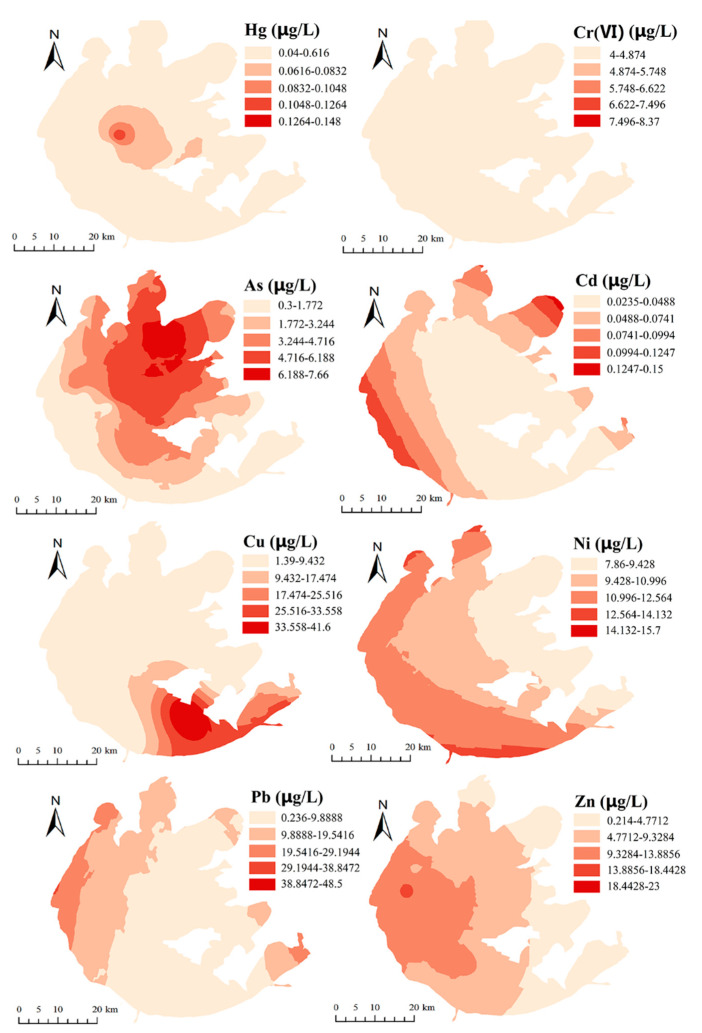
Spatial distributions of eight metals in the surface water of Taihu Lake.

The concentrations of eight HMs are shown in Table 3. The average concentrations of the metals in descending order were Pb (17.69 ± 14.79) > Ni (10.53 ± 2.03) > Zn (6.50 ± 4.85) > Cr(VI) (6.43 ± 1.95) > Cu (5.92 ± 7.41) > As (4.19 ± 1.78) > Hg (0.11 ± 0.04) > Cd (0.07 ± 0.03) µg∙L^−1^. Generally, the content of HMs in the surface water of Taihu Lake was at a relatively low level. The average contents of Cr(VI), As, Cd, Cu, and Zn in Taihu Lake were lower than the standard values for Grade I surface waters in China (GB3838-2002). The average concentration of Ni was lower than the standard value (20.0 µg∙L^−1^) for surface water stipulated in China. The average concentration of Pb in the surface water of Taihu Lake was lower than the standard value for Grade III surface waters in China (GB3838-2002), and the average concentration of Hg was lower than the standard value for Grade IV surface waters in China (GB3838-2002). As shown in Figure 2, the spatial distributions of As, Cd, Ni, Pb, and Zn in the surface water of Taihu Lake generally showed a decreasing trend from north to south and from west to east. High concentrations of As, Ni, Pb, Cd, and Zn were found in the northwest part of the Taihu Lake Basin. The high value of Cu concentration was located in the southeastern region of the Taihu Lake Basin. Regarding Hg (sampling sites 18 and 24) and Cr(VI) (sampling sites 2 and 11), only two points were detected.

### 3.2. Concentrations and Distribution of HMs in the Surface Sediments

The distributions of HMs in Taihu Lake sediments are shown in Table 4. The average concentrations of the metals in descending order were Zn (116.4 ± 65.27) > Ni (43.75 ± 20.97) > Pb (36.36 ± 9.10) > Cu (34.20 ± 20.34) > As (13.77 ± 13.75) > Cr(VI) (1.85 ± 2.72) > Cd (0.42 ± 0.20) > Hg (0.08 ± 0.05) mg∙kg^−1^. Overall, the eight HMs were detected to varying pollution degrees in the sediments, indicating that the HMs were ubiquitous in the sediments of the Taihu Lake area. The average content of Cr(VI) in Taihu Lake sediments was lower than the background value (86.00 mg∙kg^−1^), while the levels of other elements, i.e., Hg, As, Cd, Cu, Ni, Pb, and Zn, were higher than their background values [21] (Table 4), being 110.53%, 36.34%, 425.00%, 23.47%, 14.53%, 47.80%, and 57.30%, respectively.

**Figure 3:**
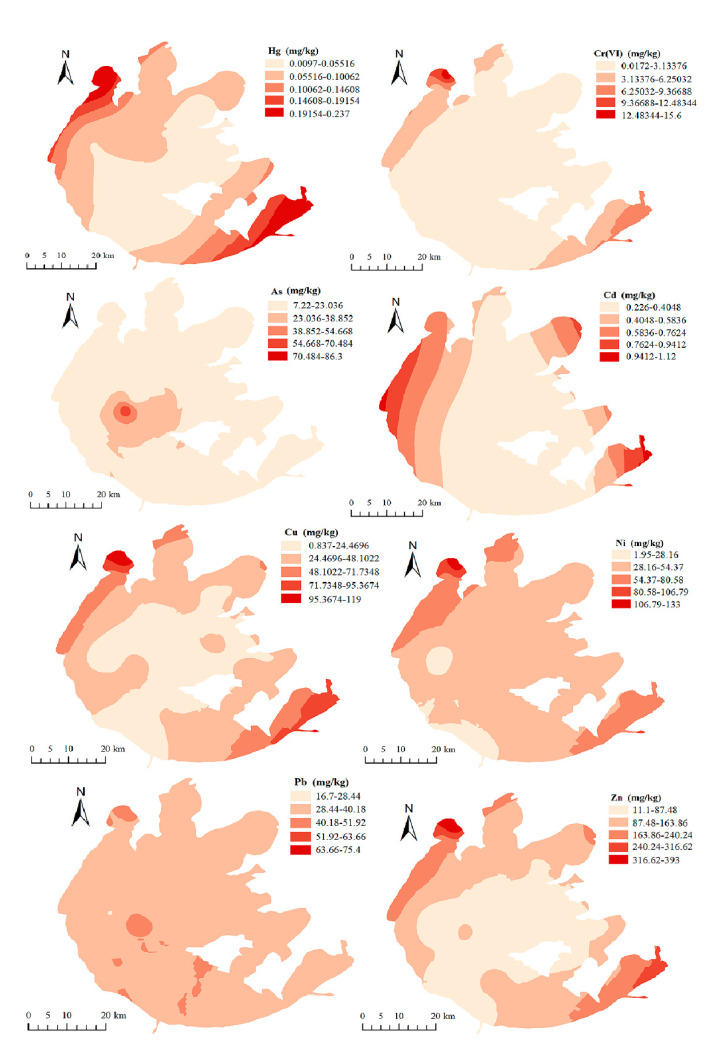
Spatial distributions of heavy metals in the sediments of Taihu Lake.

The spatial distribution of HMs in the sediments in the study area is shown in Figure 3. The maximum concentrations of Hg, Cr(VI), Cu, Ni, and Zn were 24, 780, 141.67, 70, and 35.41 times the minimum concentrations, respectively. This indicated that there was significant spatial variation in the accumulation of HMs in the sediments of Taihu Lake. Ni and Zn were the most abundant elements in the sediments of Taihu Lake, with maximum values of 133 and 393 mg∙kg^−1^, respectively. Relatively high concentrations of Hg, Cr(VI), Cd, Cu, Ni, Pb, and Zn were found in the northwest part of the lake. The results were consistent with those of a previous study. Niu et al. [11] also found that the distribution areas of HMs with higher concentrations were mainly the north bays, namely Zhushan Bay, Meiliang Bay, and Gonghu Bay. The heavy pollution of HMs in the bays of northern Taihu Lake mainly originated from the sewage discharge from urban development, while industrial emissions could be the main pollution source for the western Taihu Lake [29]. Agricultural activities, industrial emissions, and traffic transportation were the main sources of heavy metals pollution in Taihu Lake. Furthermore, the release from natural parent crustal rocks and change synchronously in regional geochemical processes may produce the baseline concentrations of metals in surface sediment [30]. According to the result of Niu et al. [13], complex industrial activities are the main sources of heavy metals in Taihu Lake sediment, contributing 64.9% of the total heavy metal concentrations. Agricultural sources, transportation sources, and natural sources have contributed 22%, 6.6%, and 6.5%, respectively. 

### 3.3. Potential Ecological Risk of HMs in the Fresh Surface Water

In this study, the risk quotient (RQ) method was used to characterize the risk of eight HMs in the surface water of Taihu Lake. The aquatic life criteria of HMs for Chinese native freshwater organisms and the corresponding RQs are shown in Table 5. The acute RQs of eight HMs were all <1, indicating no acute hazardous effects on aquatic organisms. From the perspective of long-term pollution, the chronic RQs of Cu, Ni, and Pb were 2.27, 2.44, and 3.47, respectively. This indicates that aquatic organisms living in the surface water of Taihu Lake may be threatened by Cu, Ni, and Pb for long-term exposure. This finding is consistent with the previous report that Cu, Zn, Cr, Ni, and Pb pose risks to native aquatic species in the surface water of Taihu Lake [12]. Therefore, the ecological risk of Cu, Ni, and Pb—but not other priority toxic metals—should receive more attention and be monitored periodically on the surface of Taihu Lake.

### 3.4. Potential Ecological Risk of HMs in the Surface Sediments

#### 3.4.1. Ecological Risk for Individual Metals

**Figure 4:**
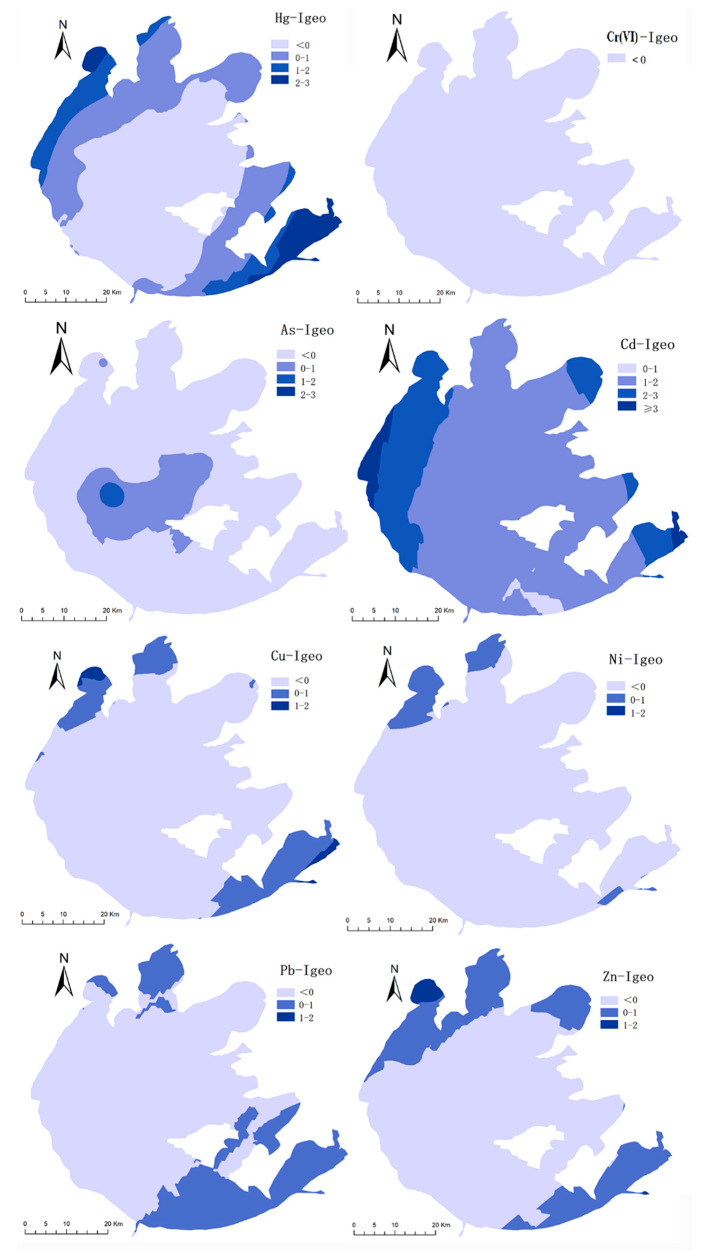
Spatial distributions of the geo-accumulation index (I*_geo_*) of heavy metals in sediments.

The average values of I*_geo_* for HMs in the surface sediments of Taihu Lake area in descending sequence were Cd (1.69) > Hg (0.19) > Pb (−0.06) > Zn (−0.13) > As (−0.37) > Cu (−0.55) > Ni (−0.59) > Cr(VI) (−6.88). The average I*_geo_* values of Hg and Cd were greater than zero, indicating that the presence of Cd and Hg in sediments poses a certain risk to aquatic organisms. The pollution degree of Cd was moderate, and the pollution degree of Hg was from no to moderate pollution. In this study, the Taihu Lake area was divided into three regions, the northern part of Taihu Lake (including sampling points 1–14, 20) near Meiliang Bay, the southwestern region (including sampling points 15–19, 21, 27–30) and the eastern region (containing sampling points 22–26). The average I*_geo_* of Cd was 1.92 in the sediments of the northern part of Taihu Lake, indicating that the occurrence of Cd was at the level of moderate pollution (Figure 4). The average I*_geo_* for the metals Hg (0.66) and Zn (0.36) were between 0 and 1, indicating no to moderate pollution of Hg and Zn in the northern part of Taihu Lake. Additionally, the average I*_geo_* values of Cr(VI) (−5.94), As (−0.58), Cu (−0.03), Ni (−0.12), and Pb (−0.01) in the sediments of northern Taihu Lake indicated that there was no pollution from these metals in the northern part of Taihu Lake. In the southwestern and eastern regions of Taihu Lake, the average I*_geo_* values of Cr(VI), As, Cu, Ni, Pb, and Zn were lower than zero, meaning no ecological risk posed by the sediments. The average I*_geo_* of Hg was 0.06 in the eastern part of Taihu Lake, indicating a moderate ecological risk of Hg. As illustrated in Figure 4, Cd generally presents a moderate risk in the sediments of the southwest and eastern parts of Taihu Lake, with the relatively higher average I*_geo_* value calculated to be 1.38 and 1.80, respectively. 

**Figure 5:**
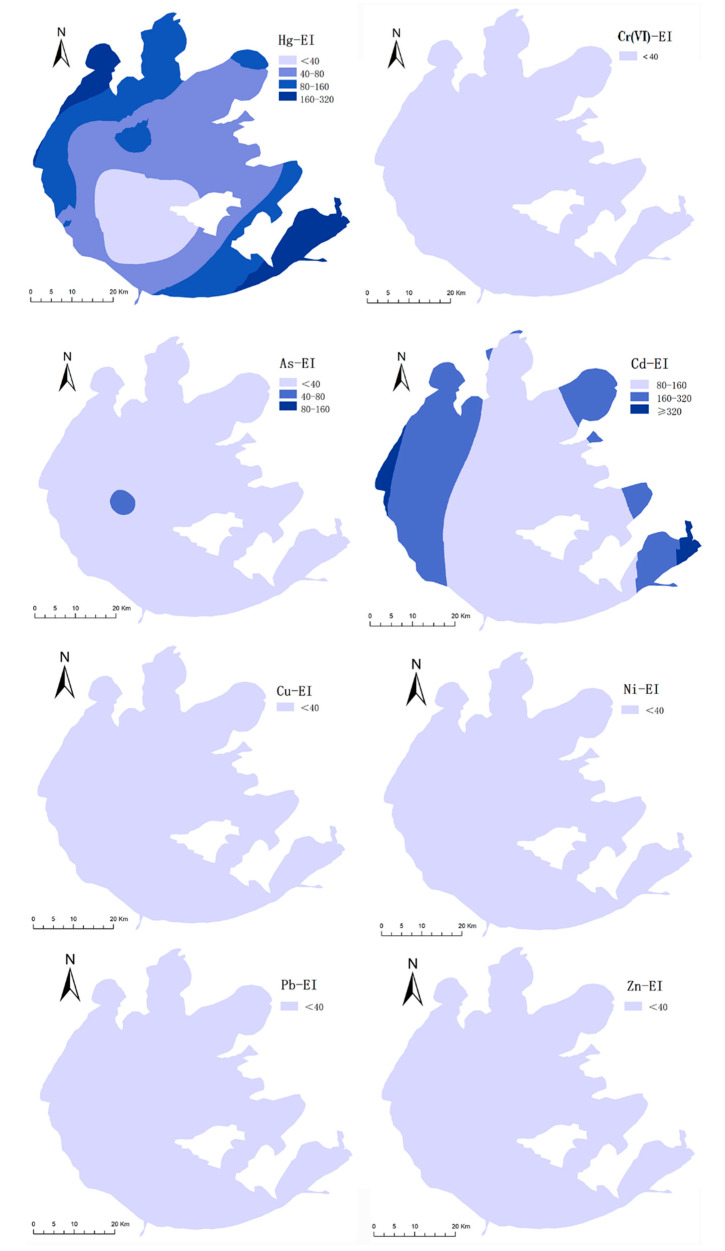
Spatial distributions of EI index for individual heavy metals in sediments.

The PI values of Hg, Cr(VI), As, Cd, Cu, Ni, Pb, and Zn in the study area were 0.26–6.24, 0.00–0.18, 0.71–8.54, 2.83–14.00, 0.03–4.30, 0.05–3.48, 0.68–3.07, and 0.15–5.31. The PI values of Cd in the sediment samples were greater than 2.0. This finding suggests that the sediments of Taihu Lake were strongly contaminated with Cd. The EI values of eight HMs in the sediments of Taihu Lake were in the order Cd > Hg > As > Pb > Cu > Ni > Zn > Cr(VI). The exposure of Cd and Hg in the sediments constituted a considerable risk, with average EI values of 158.21 and 80.27, respectively. The average EI values of Cr(VI), As, Cu, Ni, Pb, and Zn were less than 40, indicating low risk levels. As illustrated in Figure 5, the ecological risk of Cd in the sediments of the northern part of Taihu Lake (EI = 186.45) was higher than in the southwestern (EI = 143.78) and eastern areas (EI = 102.38). The EI values of Hg were 104.24 in the northern region and 52.52 and 63.92 in the southwestern and eastern regions, respectively. Therefore, Cd and Hg present considerable hazards in the sediments of the northern region of Taihu Lake. A previous study confirmed that the surface sediment of Taihu Lake was found to be most seriously polluted with Cd with higher values of I*_geo_* and RI, based on the data collected from the peer-reviewed literature through ISI Web of Science, China National Knowledge Infrastructure (CNKI) and Wan Fang Data between 2000 and 2018 [13]. In the northern Taihu Lake, toxic metals are typically discharged from dyeing, paper, ceramics, and chemical industries, which have accelerated the economic development in the past two decades. In our study, the “hot spot” of metal pollution was identified to be the northern Taihu Lake according to the pollution status of HMs in the surface water and sediment. 

#### 3.4.2. Ecological Risk for the Combined Pollution of HMs

**Figure 6:**
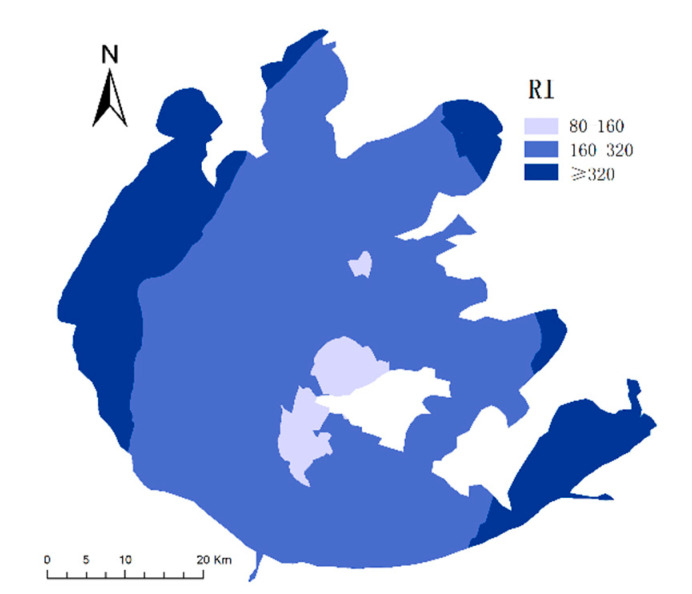
Spatial distribution of the ecological risk index (RI) of heavy metals in sediments in the study areas.

The PLI values for the surface sediments of Taihu Lake ranged from 0.23 to 2.65, with an average value of 0.92, and 23.33% of the sampling points had PLI values between 1 and 2, indicating that there may be moderate contamination. We also found that there was only one point with a PLI value >2, located at sampling point 11 in the northern region of the Taihu Lake area. The RI values for eight HMs in the surface sediments of Taihu Lake ranged from 157.70 to 569.63. The average RI value was calculated to be 273.03, which was >150, indicating that the eight HMs posed a moderate ecological risk. As illustrated in Figure 6, the RI value in northern Taihu (325.95) was higher than those of the southwestern region (231.45) and the eastern region (197.46). This also confirmed that the sediments in the northern Taihu Lake had a high level of ecological risk posed by the HMs, while the southwestern and eastern regions had moderate levels of ecological risk. Li et al. [29] also reported that northern Taihu Lake was identified as a heavy pollution region suffering from polycyclic aromatic hydrocarbons (PAHs) and HMs. 

### 3.5. Potential Risk of HMs to Human Health

Taihu Lake, investigated in this study, is located in Jiangsu Province, and thus the exposure parameters for residents living in Jiangsu Province were used to evaluate the health risks of the HMs. The average body mass, drinking water intake, and consumption of fish for the native residents of Jiangsu Province were 63.2 kg, 2.334 L∙d^−1^, and 164.4 L∙d^−1^, respectively, according to the “Exposure Factors Handbook of the Chinese Population” [37]. The results regarding non-carcinogenic HQs of Hg, Cd, Cu, and Zn and carcinogenic R of Cr(VI), As, Pb, and Ni through drinking water intake and fish consumption are listed in Table 6 and Table 7, respectively. As shown in Table 6, the values of HQ*_W_* and HQ*_F_* for Hg, Cd, Cu, and Zn were lower than 1.0, suggesting that drinking water intake and fish intake posed acceptable risks to human health. The THQ for individual metals decreased in the following sequence: Hg ≈ Zn > Cd > Pb. The non-carcinogenic Total THQ was calculated to be 0.369 in this study, indicating that the non-carcinogenic risk posed by Hg, Cd, Cu, and Zn in the fresh surface water was acceptable.

The carcinogenic risk of Cr(VI), As, Pb, and Ni through drinking water was acceptable, with R__W__ values between 10^−4^–10^−6^ (Table 7). The intake of Cr(VI) and As through fish consumption posed a potential carcinogenic risk to the health of native residents, with R__F__ values of 1.67 × 10^−3^ and 4.89 × 10^−3^ for Cr(VI) and As, respectively. Furthermore, the R*_F_* values of Cr(VI), As, and Pb were higher than the corresponding R*_W_* values, indicating that the carcinogenic risk through fish intake was higher than through water intake. The total carcinogenic risk (TR) of Cr(VI), As, and Ni through drinking water and fish consumption were 1.79 × 10^−3^, 5.12 × 10^−3^, and 1.13 × 10^−3^, respectively, values that indicated potential health risks. Therefore, the levels of toxic metals of Cr(VI), As, and Ni in edible organisms such as fish should be monitored periodically. The total carcinogenic risk (TR) for Pb through drinking water and fish consumption was acceptable.

When determining the environmental risks of HMs in surface water and sediments, some uncertainty exists. The uncertainty originates from the variation in factors such as the collected aquatic life criteria, the method of screening organisms, the selection of risk assessment models, and some unknown factors [38]. In the present study, the nationally recommended chronic and acute aquatic life criteria of Cd were used to assess the ecological risk of Cd in the surface water of Taihu Lake. However, the freshwater aquatic life criteria for other HMs that have not been released by the MEE of China were retrieved from peer-reviewed studies. The toxicity data based on different toxicity endpoints from different studies could result in uncertainty in the ecological risk analyses. Furthermore, the BCF values of Hg, Cr(VI), As, Pb, and Ni were gained from the Risk Assessment Information System (RAIS), and the BCF of Cd, Cu, and Zn for fish in Taihu Lake were applied [38]. Due to significant variability in site-specific water quality characteristics (e.g., pH value, hardness, or DOC), geographic location, or the aquatic species distribution, the method used to obtain the data may increase the uncertainty of risk analysis. The BCF of HMs differ among various organisms. Furthermore, aquatic species that are distributed throughout different regions have different sensitivities. Therefore, to minimize uncertainty as much as possible, the BCF of typical HMs in food webs in Taihu Lake should be investigated and quantified in future studies.

## 4. Conclusions

The results of the present study indicated that certain HMs posed ecological and health risks in the surface water and sediments of Taihu Lake. The acute risks of HMs to aquatic organisms were acceptable, while the chronic risk quotients of Cu, Ni, and Pb (>1.0) were found in the surface water of Taihu Lake. The risk assessment results revealed a moderate degree of HM risk in the sediments of Taihu Lake. Moreover, the ecological risk of HMs in the northern area of Taihu Lake was found to be higher than in the southwestern and eastern regions. Cd and Hg were identified as the primary contaminants in the sediments of Taihu Lake, with higher values of I*_geo_* and PLI. The non-carcinogenic risk levels for Hg, Cd, Cu, and Zn in the residents living around Taihu Lake were acceptable. However, the ingestion of Cr(VI), As, and Ni through drinking water and fish consumption poses certain health risks and thus require greater attention. As such, it is necessary for the local government to develop clean technologies to reduce the emission of HMs into the aquatic environment and to strengthen the monitoring and management of HMs in the water, sediments, and edible organisms. Northern Taihu Lake has become a “hot spot” of pollution, which should be the focus of concern for policymakers. The control measures for sewage discharge from urban development in the “hot spots” must be more concerned.

## Figures and Tables

**Table 1:** Category of the pollution load index (PLI).

PLI Value	<1	1 ≤ PLI < 2	2 ≤ PLI < 3	PLI ≥ 3
Pollution grade	Uncontaminated	Moderately contaminated	Strongly contaminated	Very strongly contaminated

**Table 2:** Category of the potential ecological risk index EI and RI.

EI and Risk Level		RI and Potential Ecological Hazard	
EI < 40	Low risk	RI < 150	Low
40 ≤ EI< 80	Moderate risk	150 ≤ RI < 300	Moderate
80 ≤ EI < 160	Considerable risk	300 ≤ RI < 600	High
160 ≤ EI < 320	High risk	RI ≥ 600	Significantly high
EI ≥ 320	Very high risk	/	/

**Table 3:** Concentrations of heavy metals in the surface water of Taihu Lake (µg∙L^−1^).

Metal	*n*	Mean ± SD	Minimum	Maximum	Median
Hg	30	0.11 ± 0.04	0.07	0.15	0.11
Cr(VI)	30	6.43 ± 1.95	4.48	8.37	6.43
As	30	4.19 ± 1.78	0.40	7.66	4.43
Cd	30	0.07 ± 0.03	0.02	0.15	0.07
Cu	30	5.92 ± 7.41	1.39	41.60	4.61
Ni	30	10.53 ± 2.03	7.86	15.70	10.20
Pb	30	17.69 ± 14.79	0.98	48.50	15.00
Zn	30	6.50 ± 4.85	0.21	23.00	6.23

**Table 4:** Sedimentary characteristics and concentrations of heavy metals in surface sediments of Taihu Lake (mg∙kg^−1^).

	Hg	Cr(VI)	As	Cd	Cu	Ni	Pb	Zn
n	30	30	30	30	30	30	30	30
Mean ± SD	0.08 ± 0.05	1.85 ± 2.72	13.77 ± 13.75	0.42 ± 0.20	34.20 ± 20.34	43.75 ± 0.97	36.36 ± 9.10	116.40 ± 65.27
Minimum	0.01	0.02	7.22	0.23	0.84	1.95	16.70	11.10
Maximum	0.24	15.60	86.30	1.12	119.00	133.00	75.40	393.00
Median	0.07	1.22	11.00	0.35	28.95	39.20	35.15	99.05
Background values (B*_n_*) [21]	0.038	86	10.1	0.08	27.7	38.2	24.6	74

**Table 5:** Risk quotients (RQs) of heavy metals in the surface water of Taihu Lake.

	Hg	Cr(VI)	As	Cd	Cu	Ni	Pb	Zn
Mean	0.11	6.43	4.19	0.07	5.92	10.53	17.69	6.50
Chronic aquatic life criteria	0.47 [31]	25.48 [32]	56.55 [33]	0.29 [34]	2.61 [10]	4.32 [32]	5.1 [35]	25.03 [36]
Acute aquatic life criteria	1.743 [31]	56.43 [32]	84.26 [33]	6.5 [34]	19.71 [10]	164.48 [32]	131 [35]	102.33 [36]
Chronic RQs	0.24	0.25	0.07	0.24	2.27	2.44	3.47	0.26
Acute RQs	0.06	0.11	0.05	0.01	0.31	0.06	0.14	0.06

**Table 6:** Estimated non-carcinogenic hazard quotients (HQs) for Hg, Cd, Cu, and Zn from drinking water and fish consumption by the native residents of Taihu Lake.

Metal	BCF (L/kg)	RfD (mg/kg/d)	C*_F_* (mg/kg)	ADD*_W_*	ADD*_F_*	HQ*_W_*	HQ*_F_*	THQ
Hg	1000 ^a^	0.0003 ^c^	0.11	1.06 × 10^−6^	2.85 × 10^−5^	0.0135	0.095	0.109
Cd	5556 ^b^	0.0005(water) ^d^ 0.001(food) ^d^	0.39	2.59 × 10^−6^	1.01 × 10^−4^	0.0052	0.101	0.106
Cu	1051 ^b^	0.04 ^c^	6.22	2.19 × 10^−4^	1.61 × 10^−3^	0.0055	0.040	0.046
Zn	19116 ^b^	0.3 ^c^	124.25	2.40 × 10^−4^	3.22 × 10^−2^	8.0 × 10^−4^	0.107	0.108

^a^: Data from the Risk Assessment Information System (RAIS). https://rais.ornl.gov/index.html (accessed on 6 August 2022). ^b^: Data from Zuo et al. [39]. ^c^: Data from the Integrated Risk Information System (IRIS), U.S. EPA. https://www.epa.gov/iris (accessed on 6 August 2022). ^d^: Data from U.S. EPA [40].

**Table 7:** Estimated carcinogenic risk for Cr(VI), As, Pb, and Ni from drinking water and fish consumption by the native residents of Taihu Lake.

Metal	BCF (L/kg)	SF (mg/kg/d)^−1^	C*_F_* (mg/kg)	ADD*_W_*	ADD*_F_*	R*_W_*	R*_F_*	TR
Cr(VI)	200 ^a^	0.5 ^a^	1.29	2.37 × 10^−4^	3.33 × 10^−3^	1.19 × 10^−4^	1.67 × 10^−3^	1.79 × 10^−3^
As	300 ^a^	1.5 ^b^	1.26	1.55 × 10^−4^	3.26 × 10^−3^	2.32 × 10^−4^	4.89 × 10^−3^	5.12 × 10^−3^
Pb	300 ^a^	0.0085 ^a^	5.31	6.53 × 10^−4^	1.38 × 10^−2^	5.55 × 10^−6^	1.17 × 10^−4^	1.22 × 10^−4^
Ni	100 ^a^	1.7 ^a^	1.05	3.89 × 10^−4^	2.73 × 10^−4^	6.61 × 10^−4^	4.65 × 10^−4^	1.13 × 10^−3^

^a^: Data from the Risk Assessment Information System (RAIS). https://rais.ornl.gov/index.html (accessed on 6 August 2022). ^b^: Data from the Integrated Risk Information System (IRIS), U.S. EPA. https://www.epa.gov/iris (accessed on 6 August 2022).

## Data Availability

The data presented in this study are available on request from the corresponding author.

## References

[1] Zhang W., Zhang L. (2021). Distribution characteristics and pollution assessment of heavy metals in water and sediments of Aha Reservoir of Guizhou in different seasons. Chin. J. Ecol..

[2] Sheng Y. (2017). Research Progress on Risk Assessment and Migration of Heavy Metal Pollution in Water Sediments. China Res. Compre. Util..

[3] Nguyen B.T., Do D.D., Nguyen T.X., Nguyen V.N., Nguyen D.T., Nguyen M.H., Bach Q. (2019). Seasonal, spatial variation, and pollution sources of heavy metals in the sediment of the Saigon River. Vietnam. Environ. Pollut..

[4] Zhou Q., Yang N., Li Y., Ren B., Ding X., Bian H., Yao X. (2020). Total concentrations and sources of heavy metal pollution in global river and lake water bodies from 1972 to 2017. Glob. Ecol. Conserv..

[5] Gao J., Li J., Wang H., Wang Y., Wang Z., Bai F., Gao S., Cheng Y. (2009). Distribution and their pollution assessment of heavy metals in the sediments of the Yalu River Estuary and its adjacent coastal waters. Acta Oceanol. Sin..

[6] Liu E.F., Shen J., Zhu Y.X., Xia W.L., Zhu G.W. (2004). Source Analysis of heavy metals in surface sediment of Lake Taihu. J. Lake Sci..

[7] Pekey H. (2006). Heavy metal pollution assessment in sediments of the Izmit Bay, Turkey. Environ. Monit. Assess..

[8] Lu H., Yu S. (2018). Spatio-temporal variational characteristics analysis of heavy metals pollution in water of the typical northern rivers, China. J. Hydrol..

[9] Shi H., Shi X., Liu K. (2004). Oxidative mechanism of arsenic toxicity and carcinogenesis. Mol. Cell. Biochem..

[10] Zhang X., Fu W.Q., Feng C., Wu D., Zeng H., Li X. (2016). Water quality criteria and ecological risk assessment of copper in Chinese freshwaters. Environ. Eng..

[11] Niu Y., Jiao W., Yu H., Niu Y., Pang Y., Xu X., Guo X. (2015). Spatial evaluation of heavy metals concentrations in the surface sediment of Taihu Lake. Int. J. Environ. Res. Public Health.

[12] Fu Z., Wu F., Chen L., Xu B., Feng C., Bai Y., Liao H., Sun S., Giesy J.P., Guo W. (2016). Copper and zinc, but not other priority toxic metals, pose risks to native aquatic species in a large urban lake in Eastern China. Environ. Pollut..

[13] Niu Y., Jiang X., Wang K., Xia J.D., Jiao W., Niu Y., Yu H. (2020). Meta analysis of heavy metal pollution and sources in surface sediments of Lake Taihu, China. Sci. Total Environ..

[14] Sun F.H., Mu Y.S., Leung K.M.Y., Su H.L., Wu F.C., Chang H. (2021). China is establishing its water quality standards for enhancing protection of aquatic life in freshwater ecosystems. Environ. Sci. Policy.

[15] MEE (Ministry of Ecology and Environment of the People’s Republic of China) (2014). Water Quality-Determination of Mercury, Arsenic, Selenium, Bismuth and Antimony-Atomic Fluorescence Spectrometry (HJ 694—2014). https://www.mee.gov.cn/ywgz/fgbz/bz/bzwb/jcffbz/201403/t20140319_269361.htm.

[16] MEE (Ministry of Ecology and Environment of the People’s Republic of China) (2017). Water Quality-Determination of Chromium(VI)-Flow Injection Analysis(FIA) and Diphenylcarbazide Spectrometric Method (HJ 908-2017). https://www.mee.gov.cn/ywgz/fgbz/bz/bzwb/jcffbz/201801/t20180108_429323.shtml.

[17] MEE (Ministry of Ecology and Environment of the People’s Republic of China) (2016). Soil and Sediment Determination of Aqua Regia Extracts of 12 Metal Elements- Inductively Coupled Plasma Mass Spectrometry (HJ 803-2016). https://www.mee.gov.cn/ywgz/fgbz/bz/bzwb/jcffbz/201606/W020160701531772799289.pdf.

[18] MEE (Ministry of Ecology and Environment of the People’s Republic of China) (2019). Soil and Sediment-Determination of Chromium(VI)-Alkaline Digestion/Flame Atomic Absorption Spectrometry (HJ 1082-2019). https://www.mee.gov.cn/ywgz/fgbz/bz/bzwb/jcffbz/202001/t20200102_756539.shtml.

[19] Tao Y., Su H., Li H., Zhu Y., Shi D., Wu F., Sun F. (2021). Ecological and human health risk assessment of antimony (Sb) in surface and drinking water in China. J. Clean. Prod..

[20] Muller G. (1979). Heavy-metals in sediment of the Rhine-changes since 1971. Umsch. Wiss. Tech..

[21] Cheng H., Li M., Zhao C., Yang K., Li K., Peng M., Yang Z., Li F., Liu Y., Bai R. (2015). Concentrations of toxic metals and ecological risk assessment for sediments of major freshwater lakes in China. J. Geochem.Explor..

[22] Huang L., Rad S., Xu L., Gui L., Song X., Li Y., Wu Z., Chen Z. (2020). Heavy metals distribution, sources, and ecological risk assessment in Huixian Wetland, South China. Water.

[23] Tian K., Huang B., Xing Z., Hu W. (2017). Geochemical baseline establishment and ecological risk evaluation of heavy metals in greenhouse soils from Dongtai, China. Ecolog. Indicat..

[24] Tomlinson D.L., Wilson J.G., Harris C.R., Jeffrey D.W. (1980). Problems in the assessment of heavy-metal levels in estuaries and the formation of a pollution index. Helgolaender Meeresunters..

[25] Hakanson L. (1980). An ecological risk index for aquatic pollution control.a sedimentological approach. Water Res..

[26] United States Environmental Protection Agency (U.S. EPA) (1989). Risk Assessment Guidance for Superfund Volume I Human Health Evaluation Manual (Part A).

[27] United States Environmental Protection Agency (U.S. EPA) (2000). Supplementary Guidance for Conducting Health Risk Assessment of Chemical Mixtures. Risk Assessment Forum Technical Panel.

[28] United States Environmental Protection Agency (U.S. EPA) (2007). Framework for Metals Risk Assessment. Office of the Science Advisor. Risk Assessment Forum.

[29] Li Y., Wang X.P., Gong P. (2021). Combined risk assessment method based on spatial interaction: A case for polycyclic aromatic hydrocarbons and heavy metals in Taihu Lake sediments. J. Clean. Prod..

[30] Wang S.H., Wang W.W., Chen J.Y., Zhao L., Zhang B., Jiang X. (2019). Geochemical baseline establishment and pollution source determination of heavy metals in lake sediments: A case study in Lihu Lake, China. Sci. Total Environ..

[31] Zhang R., Wu F., Li H., Feng C., Guo G. (2012). Deriving aquatic water quality criteria for inorganic mercury in China by species sensitivity distributions. Acta Scientiae Circumstantiae..

[32] Du D. (2012). The Study on Water Quality Criteria of Heavy Metals Chromium and Nickel in China.

[33] Zhang J., Yan Z., Gao F., Wu J., Pei S., Zhou J., Liu Z. (2015). Development of aquatic life criteria for arsenic species and its application in Liao River. Acta Sci. Circumstantiae.

[34] MEE (Ministry of Ecology and Environment of the People’s Republic of China) (2017). The Technical Guidelines for Deriving Water Quality Criteria for the Protection of Freshwater Aquatic Organisms. http://www.mee.gov.cn/xxgk2018/xxgk/xxgk01/202003/t20200303_766970.html.

[35] Yan Z.G., He L., Gao F., Zhou J.L., Wang X.N., Wang W.L., Liu Z.T. (2013). Development premilinary applications of freshwater aquatic life water quality criteria for lead in China. Proceedings of Conference on Environmental Standards & Criteria, Environmental & Ecology Toxicology.

[36] Liu D., Li X., Fu W., Huang C., Yang H., Feng C. (2017). Water quality criteria of zinc for the protection of freshwater of organisms and its ecological risk in China. Environ. Eng..

[37] SEPA (State Environmental Protection Administration) (2013). Exposure Factors Handbook of the Chinese Population.

[38] Chen C. (2005). Ecological risk assessment for aquatic species exposed to contaminants in Keelung River, Taiwan. Chemosphere.

[39] Zuo J., Fan W., Wang X., Ren J., Zhang Y., Wang X., Zhang Y., Yu T., Li X. (2018). Trophic transfer of Cu, Zn, Cd, and Cr, and biomarker response for food webs in Taihu Lake, China. RSC Adv..

[40] United States Environmental Protection Agency (U.S. EPA) (2002). National Recommended Water Quality Criteria: 2002, Human Health Criteria Calculation Matrix.

